# HIV-1 Maturation: Lessons Learned from Inhibitors

**DOI:** 10.3390/v12090940

**Published:** 2020-08-26

**Authors:** Alex B. Kleinpeter, Eric O. Freed

**Affiliations:** HIV Dynamics and Replication Program, Center for Cancer Research, National Cancer Institute, Frederick, MD 21702, USA; alex.kleinpeter@nih.gov

**Keywords:** HIV-1, maturation, capsid, Gag, assembly, maturation inhibitor, capsid inhibitor, ALLINI

## Abstract

Since the emergence of HIV and AIDS in the early 1980s, the development of safe and effective therapies has accompanied a massive increase in our understanding of the fundamental processes that drive HIV biology. As basic HIV research has informed the development of novel therapies, HIV inhibitors have been used as probes for investigating basic mechanisms of HIV-1 replication, transmission, and pathogenesis. This positive feedback cycle has led to the development of highly effective combination antiretroviral therapy (cART), which has helped stall the progression to AIDS, prolong lives, and reduce transmission of the virus. However, to combat the growing rates of virologic failure and toxicity associated with long-term therapy, it is important to diversify our repertoire of HIV-1 treatments by identifying compounds that block additional steps not targeted by current drugs. Most of the available therapeutics disrupt early events in the replication cycle, with the exception of the protease (PR) inhibitors, which act at the virus maturation step. HIV-1 maturation consists of a series of biochemical changes that facilitate the conversion of an immature, noninfectious particle to a mature infectious virion. These changes include proteolytic processing of the Gag polyprotein by the viral protease (PR), structural rearrangement of the capsid (CA) protein, and assembly of individual CA monomers into hexamers and pentamers that ultimately form the capsid. Here, we review the development and therapeutic potential of maturation inhibitors (MIs), an experimental class of anti-HIV-1 compounds with mechanisms of action distinct from those of the PR inhibitors. We emphasize the key insights into HIV-1 biology and structure that the study of MIs has provided. We will focus on three distinct groups of inhibitors that block HIV-1 maturation: (1) compounds that block the processing of the CA-spacer peptide 1 (SP1) cleavage intermediate, the original class of compounds to which the term MI was applied; (2) CA-binding inhibitors that disrupt capsid condensation; and (3) allosteric integrase inhibitors (ALLINIs) that block the packaging of the viral RNA genome into the condensing capsid during maturation. Although these three classes of compounds have distinct structures and mechanisms of action, they share the ability to block the formation of the condensed conical capsid, thereby blocking particle infectivity.

## 1. Introduction

**Figure 1:**
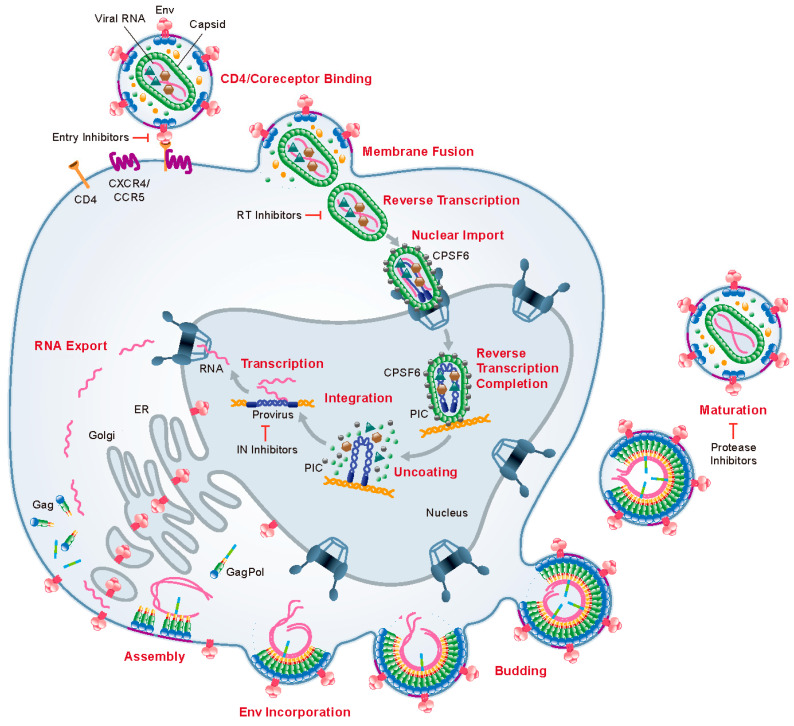
HIV-1 replication cycle. Mature virions attach to the surface of a target cell via an interaction between the gp120 subunits of viral Env trimers and host CD4 and coreceptor (CCR5 or CXCR4). The gp41 subunits of Env then trigger the fusion of the viral and host cell membranes and the capsid is deposited into the cytosol. Reverse transcription proceeds as the capsid traffics to the nuclear envelope where it is imported into the nucleus in an intact or nearly intact state. Reverse transcription is completed in the nucleus, followed by uncoating of the capsid and integration of the viral DNA into the host genome. Transcription of the proviral DNA leads to the expression of HIV-1 proteins including the Gag and GagPol polyproteins. These structural proteins are targeted to the inner leaflet of the plasma membrane where they form hexameric subunits that assemble into a curved, membrane-associated lattice. Viral genomic RNA dimers are recruited to these sites of assembly and Env trimers are incorporated into the budding particles. Membrane scission and particle release are facilitated by endosomal sorting complexes required for transport (ESCRT) recruitment mediated by the Gag-p6 domain. Concomitant with particle release, protease (PR), which is packaged into virions as part of the GagPol polyprotein, cleaves the Gag and GagPol polyproteins into their individual mature proteins. The newly liberated CA monomers then form hexamers and pentamers that assemble into the mature capsid, resulting in a mature, infectious particle. The site of action of FDA-approved antiretroviral drugs (ARVs) is indicated. Additional details are provided in the main text. Viral proteins are not shown to scale.

HIV-1 replication (Figure 1) begins with virus attachment and fusion of the viral envelope with the target cell plasma membrane mediated by the viral envelope glycoprotein (Env) and the CD4 receptor and coreceptor (CXCR4 or CCR5) [1]. The viral capsid containing the HIV-1 genome and replicative enzymes is then released into the cytoplasm where the viral reverse transcriptase (RT) enzyme transcribes the viral RNA genome into a double-stranded DNA copy. The trafficking of these reverse transcription complexes (RTCs) to the nuclear envelope for entry into the nucleus proceeds along the host microtubule network [2]. In the nucleus, the viral integrase (IN) enzyme directs the integration of the viral DNA into transcriptionally active sites within the host chromatin. Importantly, nuclear entry and integration rely on interactions with host proteins such as nucleoporins 153 and 358 (Nup153 and Nup358), the cleavage and polyadenylation specificity factor 6 (CPSF6), and the lens epithelium-derived growth factor/p75 (LEDGF/p75) [2]. The integrated proviral DNA is then transcribed into viral mRNAs and full-length genomic RNA, and the Gag and GagPol polyproteins are translated at a ratio of ~20:1 [3]. The Gag and GagPol polyproteins are targeted to the inner leaflet of the plasma membrane (PM) where they assemble into an immature hexameric lattice, inducing curvature in the PM. During its trafficking through the Golgi apparatus, the Env glycoprotein precursor, gp160, is cleaved by host furin or furin-like proteases to generate the surface glycoprotein subunit gp120 and the transmembrane glycoprotein gp41. Viral genomic RNA dimers are packaged by the assembling Gag lattice and heterotrimeric gp120/gp41 Env complexes are incorporated into the membrane at the sites of assembly [4]. Immature virus particles are released from the host cell via membrane scission directed by the host endosomal sorting complexes required for transport (ESCRT) machinery [5]. Concomitant with release, Gag polyproteins are cleaved into their constituent mature proteins by the viral protease (PR). Like the other viral enzymes RT and IN, PR is packaged into virions as part of the GagPol polyprotein. PR-mediated Gag cleavage triggers the disassembly of the membrane-bound immature Gag lattice, resulting in the assembly of the mature conical capsid from fully processed capsid protein (CA) [6]. The processes including cleavage of Gag followed by structural rearrangement within the virion are collectively referred to as maturation. Proper maturation, which is essential to virion infectivity, requires the packaging of two single-stranded copies of the viral RNA genome, together with RT and IN, into the nascent capsid [7]. (Note that the term “capsid” applies to both the mature capsid protein (CA) and the capsid core. Here, we adhere to the convention of referring to the protein as CA and the core as the capsid).

Presently, there are five classes of FDA-approved antiretroviral drugs (ARVs), most of which target early steps in the virus replication cycle (Figure 1). Entry inhibitors block Env-mediated viral entry via multiple mechanisms. The fusion inhibitor enfuvirtide binds to gp41 to prevent fusion of the virus and host cell membranes during entry. The CCR5 antagonist maraviroc blocks HIV-1 entry by binding the CCR5 coreceptor on the surface of host cells, thereby preventing the interaction between gp120 and CCR5. Similarly, the post-attachment inhibitor ibalizumab binds to CD4 and inhibits entry by blocking the engagement of the coreceptors CCR5 or CXCR4 by gp120. Nucleoside RT inhibitors (NRTIs) and non-nucleoside RT inhibitors (NNRTIs) target RT either by binding directly to an allosteric site on the enzyme (NNRTIs) or providing a chain-terminating substrate during reverse transcription (NRTIs). IN strand transfer inhibitors (INSTIs) bind the IN/viral DNA complex, effectively preventing viral DNA integration. Finally, PR inhibitors (PIs) act late during the virus replication cycle by blocking the proteolytic cleavage of the Gag and GagPol polyprotein precursors, resulting in a defect in virus particle maturation. cART regimens typically include three drugs from at least two different classes of inhibitor (usually two NRTIs and one NNRTI, PI, or INSTI). The success of cART in treating people living with HIV is mainly due to its ability to suppress the replication of the virus and reduce the viral load to levels that are extremely low and often undetectable in clinical assays. However, cART is not curative and the development of HIV-1 resistance leading to virologic failure is an increasingly significant problem. This highlights the need for continued development of new ARVs that exploit novel targets to inhibit HIV-1 replication [8,9].

**Figure 2:**
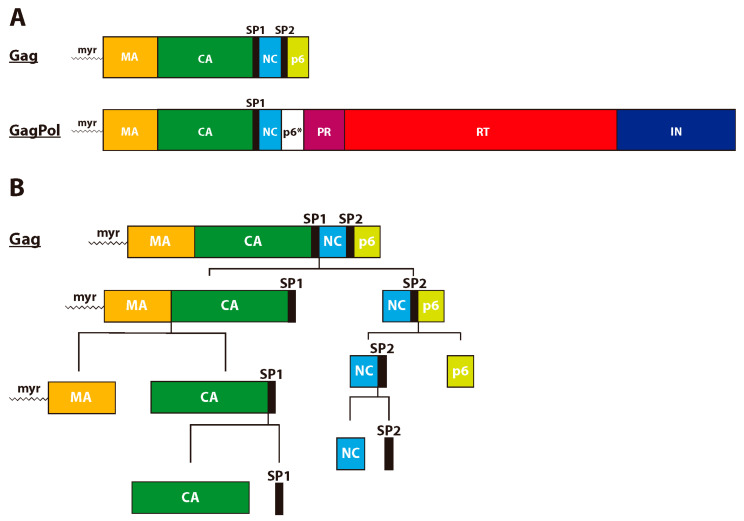
Organization of the Gag and GagPol polyproteins. (**A**) The Gag and GagPol polyproteins are expressed at roughly a 20:1 ratio during the late stages of HIV-1 infection as the result of a -1 frameshift during Gag translation. The Gag polyprotein contains the matrix (MA), capsid (CA), nucleocapsid (NC), and p6 domains with two spacer peptides (SP1 and SP2) flanking NC. The incorporation of GagPol into the Gag lattice provides immature virions with the necessary amount of viral enzymes (PR, reverse transcriptase (RT) and integrase (IN)) required for viral maturation, reverse transcription, and integration. p6* indicates the region of the *pol* open reading frame that overlaps with Gag-p6. The myristic acid moiety covalently attached to the N-terminus of the MA domain is indicated (myr). (**B**) The order of proteolytic cleavage events during maturation is depicted for the Gag polyprotein.

**Figure 3:**
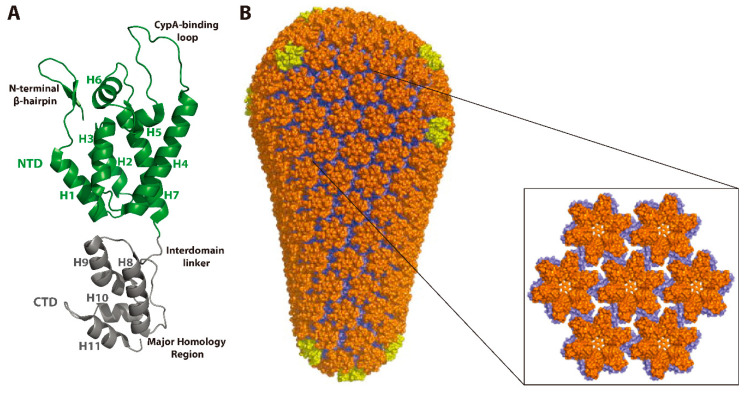
The HIV-1 capsid. During maturation, proteolytic processing of the Gag polyprotein liberates the mature CA protein. (**A**) The monomeric CA (PDB accession 2M8N) is a predominantly α-helical protein with N- and C-terminal domains (CA-NTD and CA-CTD, respectively) connected by a flexible interdomain linker region. Individual α-helices are indicated (H1-H11) and the locations of key structural elements (e.g., the N-terminal β-hairpin, CypA-binding loop, interdomain linker, and major homology region) are labeled. (**B**) In the final stage of maturation, CA monomers multimerize into hexameric (orange) and pentameric (yellow) subunits that assemble into a conical structure exhibiting fullerene geometry. This protein superstructure, known as the capsid, contains approximately 250 CA hexamers and exactly 12 CA pentamers. The incorporation of pentameric subunits is concentrated at either end of capsid to provide the curvature necessary to close this structure. The CA-NTD is labeled in orange and the CA-CTD in blue. Panel B images are reprinted with permission from the authors of references [6] and [33] and from Cell Press and Nature Publishing Group.

The HIV-1 Gag polyprotein precursor (Pr55^Gag^, or Gag) plays a key role in the assembly, release, and maturation of virus particles (reviewed in references [7,10,11,12,13,14]). Gag contains the matrix (MA), CA, nucleocapsid (NC), and p6 domains, along with two spacer peptides, SP1 and SP2, which flank the NC domain (Figure 2A). The MA domain targets Gag to the plasma membrane where the CA domain promotes Gag multimerization and ultimately the assembly of the immature Gag lattice. The NC domain also contributes to Gag assembly via protein–RNA interactions responsible for the recruitment of the viral RNA genome [4]. The p6 domain recruits the host ESCRT machinery to facilitate membrane scission and virus release. Viral maturation begins with the proteolytic processing of Gag by PR and proceeds sequentially based on differences in the cleavage rate at each site (Figure 2B). The first cleavage event occurs between SP1 and the NC domain. Next, the MA-CA and SP2-p6 junctions are cleaved, followed by cleavage at the NC-SP2 site [15,16,17]. The final cleavage event occurs between the CA domain and SP1, liberating the CA protein, which then assembles into the conical-shaped mature capsid consisting of ~250 CA hexamers and 12 CA pentamers (Figure 3). Proper Gag processing is required for viral maturation and infectivity and the final CA-SP1 cleavage step is particularly crucial. In fact, non-cleavable CA-SP1 mutants expressed in trans with wild-type CA-SP1 exert a dominant-negative effect on proteolytic maturation, particle infectivity, and capsid formation [18,19]. The importance of the CA-SP1 boundary in maturation is determined by the presence of a key structural element within this region. Below, we will discuss the role of this structural element and others within CA during maturation and capsid formation.

**Figure 4:**
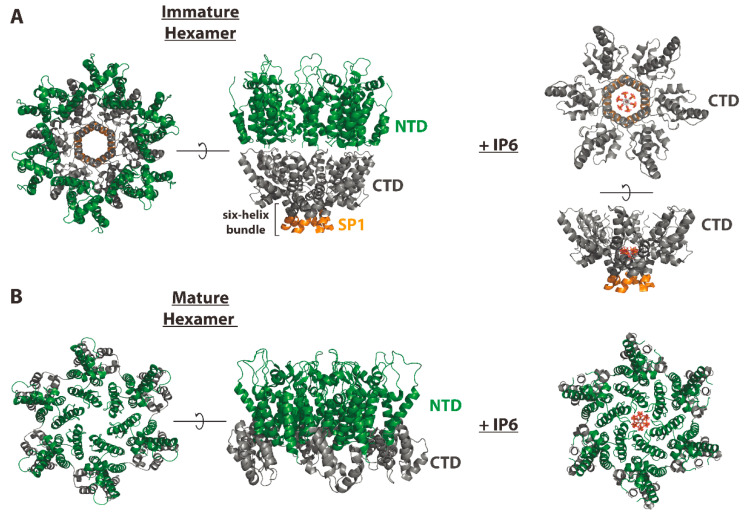
IP6 binds to immature Gag hexamers and mature CA hexamers. (**A**) The CA-CTD (gray) and SP1 (orange) of Gag facilitate the formation of hexamers that assemble into the immature Gag lattice. IP6 promotes Gag assembly by binding at the center of Gag hexamers just above the six-helix bundle, a key structural element consisting of the C-terminal amino acid residues of CA and the N-terminal amino acid residues of SP1. (**B**) Mature CA hexamer formation is driven primarily by CA-NTD/CA-NTD interactions, though CA–NTD/CA–CTD interactions also contribute. IP6 promotes mature hexamer and capsid assembly by binding to a central pore within CA hexamers responsible for facilitating nucleotide import (figures generated using PDB accessions 4USN, 5I4T, 6BHR, and 6ES8).

CA consists of two predominantly α-helical, independently folded subdomains connected by a flexible interdomain linker (Figure 3A). The CA N-terminal domain (CA-NTD) promotes the assembly of the mature CA hexamer and pentamer subunits that comprise the fully assembled capsid (Figure 3B). Conversely, the CA C-terminal domain (CA-CTD) facilitates the assembly of Gag hexamers to form the immature Gag lattice during assembly. A key structural element driving the hexamerization of Gag is a dynamic, α-helical region present at the junction of the CTD and SP1 [20]. This “junction-helix” forms a six-helix bundle in the context of unprocessed Gag, stabilizing immature Gag hexamers [21,22] (Figure 4). Proteolytic cleavage at the CA-SP1 junction during maturation disrupts this junction-helix and destabilizes Gag hexamers, leading to disassembly of the immature lattice. While immature lattice assembly is absolutely dependent on the CA-SP1 junction-helix and six-helix bundle formation, cleavage-deficient CA-SP1 can assemble into mature-like CA hexamers in vitro [20] and in virions [23] without undergoing disassembly [24]. Importantly, the CA-SP1 junction is not folded and the six-helix bundle is not present in these assemblies [20,23], suggesting that loss of the six-helix bundle may support the structural transition of CA during maturation. A recent cryo-electron tomography (cryo-ET) study showed that proteolytic cleavage at either CA terminus can be sufficient for some mature-like CA assembly to occur in virions. However, complete Gag processing was still required for capsid formation [23]. Upon MA-CA cleavage, the first 12 residues of the CA-NTD fold into an N-terminal β-hairpin (Figure 3) facilitated by a salt bridge that forms between the CA Pro-1 and Asp-51 side chains [25,26]. This β-hairpin is a key structural feature of CA in the context of fully assembled capsids. However, the formation of this β-hairpin is not necessary for the formation of a mature-like CA lattice and can be tolerated in the context of an immature-like lattice during maturation in virions [23]. These data again point to six-helix bundle destabilization as the “switch” that drives structural maturation of CA.

While the structural conversion of CA is an important step during maturation, the ultimate objective is the formation of a metastable capsid carrying the genome and viral enzymes necessary for the productive infection of a new target cell. A crucial yet often-overlooked aspect of maturation is the encapsidation of the viral genomic RNA within the capsid. After Gag processing, viral RNA and NC condense to form an electron-dense complex of ribonucleoprotein (RNP) that is visible within the capsid by electron microscopy (EM) [27]. The processes driving the encapsidation of this RNP during maturation are not well understood. However, mutations in the viral IN that result in particles exhibiting eccentric cores in which the RNP is present outside of the capsid have been reported [28,29,30,31]. The assembly and stability of the capsid shell is driven by three sets of intermolecular protein–protein interactions between CA subunits: (1) intrahexameric NTD–NTD contacts between individual CA molecules stabilize the hexamers and pentamers that function as the building blocks of the capsid, (2) intrahexameric NTD–CTD contacts between adjacent CA molecules further stabilize the individual hexamers and pentamers, and (3) interhexameric CTD–CTD contacts participate in dimeric and trimeric interactions to link the individual hexamers and pentamers [32,33,34] (Figure 3B). The stability of the capsid is important not only for preserving its structure inside the virion but also for key steps early in the virus replication cycle. Although early models proposed that the capsid uncoats soon after membrane fusion, accumulating evidence now suggests that upon release into the cytoplasm, the capsid remains intact, or nearly intact, as it traffics to the nuclear envelope [35,36] and into the nucleus [37,38] (Figure 1). Optimal capsid stability is crucial for viral infectivity, as CA mutations that stabilize or destabilize cores relative to wild-type exhibit drastically reduced reverse transcription and particle infectivity [39]. Furthermore, capsids are dependent on numerous host proteins to facilitate nuclear import [35,40,41,42,43] and viral DNA integration [44,45,46]. Importantly, many of these interactions occur at critical intermolecular interfaces on the capsid and are therefore dependent on the presence of an intact (or at least semi-intact) hexameric CA lattice [47,48].

### IP6 Promotes the Assembly of the Immature Gag Lattice and Mature Capsids

Recent studies have shown that inositol hexakisphosphate (IP6), an abundant cellular polyanion, is a critical host cofactor in HIV-1 replication [49,50,51]. In vitro, IP6 potently enhances the assembly of HIV-1 Gag into immature virus-like particles via an interaction with two electropositive lysine rings in the core of the Gag hexamer, just above the six-helix bundle [49] (Figure 4). Mutation of either lysine ring leads to dramatic defects in virus production, infectivity, and IP6 packaging [49,50]. Furthermore, it has been demonstrated that a specific lysine-ring mutant that does retain some virus production exhibits unstable capsids that are highly defective in reverse transcription and infectivity. Interestingly, genetic knockout of IP6-synthesizing enzymes in producer cells leads to decreased viral production but no effect on infectivity or IP6 packaging, suggesting that cellular IP6 levels are rate-limiting for virus production but do not affect particle integrity [50]. IP6 also enhances mature capsid assembly and core stability leading to increased efficiency of encapsidated reverse transcription. Binding of IP6 to capsids is mediated by an electropositive pore that binds and facilitates the transport of deoxynucleotide triphosphates (dNTPs) into the capsid [51,52]. These data suggest a model in which IP6 facilitates assembly of the immature Gag lattice by stabilizing the six-helix bundle and is effectively packaged into virions. Proteolytic cleavage of Gag then releases IP6 from this binding site, allowing it to promote the assembly and stability of the mature capsid. Studies aimed at evaluating this model and refining our understanding of IP6′s role during HIV-1 replication are ongoing.

## 2. MIs That Block CA-SP1 Processing

### 2.1. First-in-Class MI BVM

**Figure 5:**
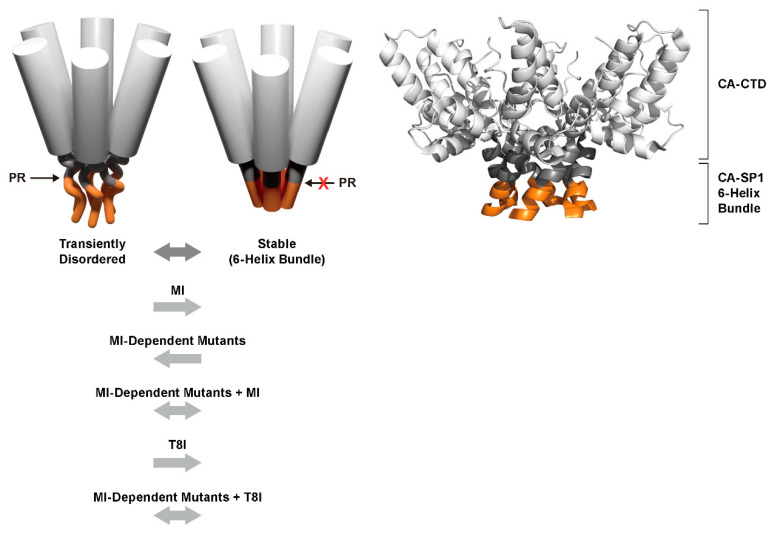
Current model for maturation inhibitor (MI) antiviral activity. The CA-SP1 six-helix bundle region (PDB accession 6BHR) exists in a dynamic equilibrium between stable and transiently disordered states. MI treatment stabilizes the bundle, shifting the equilibrium toward a more rigid structure. Because the CA-SP1 cleavage site is located inside the six-helix bundle, stabilization of the bundle denies PR access to the cleavage site and CA-SP1 processing and maturation are inhibited. MI-dependent mutants selected during propagation of HIV-1 in the presence of MIs shift this equilibrium in the opposite direction and are often assembly defective. MI treatment of these mutants restores this equilibrium and facilitates replication. The SP1-T8I mutation also stabilizes the six-helix bundle and thus phenocopies MIs in blocking CA-SP1 processing and maturation. Finally, the combination of a MI-dependent mutant with T8I (which was selected for during the passage of MI-dependent mutants in the absence of MIs) restores the native six-helix bundle equilibrium and rescues the replication defect of the MI-dependent mutants.

In 1994, a screen for anti-HIV-1 compounds identified a plant-derived triterpenoid molecule, betulinic acid, as a weak (low-µM) inhibitor of HIV-1 replication [53]. Chemical modification of betulinic acid led to the discovery of 3-O-(3′,3′-dimethylsuccinyl) betulinic acid (referred to as DSB, PA-457, and, ultimately, bevirimat (BVM)), which displayed significantly increased antiviral potency and therapeutic index relative to betulinic acid [54] (Table 1). A subsequent study demonstrated that BVM acts late in the virus replication cycle [55], and a combination of biochemical and EM analyses revealed that this compound blocks HIV-1 maturation by inhibiting a late step in the Gag processing cascade, the conversion of CA-SP1 to CA [56,57,58]. Consistent with CA-SP1 processing as the target for inhibition by BVM, selection experiments identified a panel of resistance mutations flanking the CA-SP1 cleavage site [56,57,59]. The ability of BVM to block CA-SP1 processing requires Gag assembly [56,60], suggesting that the binding site is present on multimeric (assembled) Gag but not on monomeric Gag. Binding assays with radiolabeled BVM and mass spectrometry analyses showed that BVM binds immature but not mature HIV-1 particles, consistent with the hypothesis that the binding site, putatively located in the vicinity of the CA-SP1 junction, is destroyed upon PR-mediated cleavage at this site [61]. Mutations at the CA-SP1 junction that confer resistance to BVM significantly reduce binding of radiolabeled compound to immature particles [62,63], suggesting that it binds directly to the CA-SP1 junction. Cross-linking analysis with a photoaffinity analog of BVM confirmed that the compound binds the CA-SP1 region [64], as has been demonstrated more recently in structural studies (see below). Interestingly, some cross-linking of this analog to a region of CA known as the major homology region (MHR) (Figure 3A) was also observed, implicating the MHR in MI activity [64]. BVM resistance mutations in SP1 residue 3 were highly defective in assembly and replication but were partially rescued in the presence of the compound [59], demonstrating that BVM binding to these Gag mutants offsets the effects of the mutations (Figure 5).

### 2.2. Identification and Characterization of PF-46396 (PF96), a Structurally Distinct Inhibitor of CA-SP1 Processing

In 2009, Pfizer reported a pyridone-based compound, PF96, that, like BVM, blocks CA-SP1 processing [65]. This compound was initially identified in a mechanism-blind screen for inhibitors of HIV replication. Although both BVM and PF96 block CA-SP1 processing [65,66], they are structurally distinct (Table 1). If the two compounds bind to different sites in Gag, one might predict that they would display additive or synergistic antiviral activity. Waki and colleagues instead observed that PF96 antagonizes BVM activity. This result suggests that the two compounds share at least a portion of the same binding pocket. To investigate this question further, Waki et al. performed in vitro selections for PF96 resistance [66]. Resistance-conferring mutations were identified not only in the vicinity of the CA-SP1 cleavage site where BVM-resistance mutations arose, but also far upstream in CA. A cluster of mutations was mapped to the MHR; in the absence of PF96, these mutants were highly assembly deficient and replicated poorly, whereas in the presence of PF96, these defects were rescued [66]. Analysis of a panel of PF96 analogs lead to the identification of compounds that could rescue PF96-dependent mutants, yet were devoid of antiviral activity [67]. The compound-dependent MHR mutants, when propagated in the absence of PF96, reverted through the acquisition of a second-site compensatory mutation at SP1 residue 8 (the SP1-T8I mutation). The SP1-T8I mutation also arose in the context of the BVM-dependent mutants containing substitutions at SP1 residue 3 [59]. Strikingly, the SP1-T8I mutation on its own phenocopies MI binding by blocking CA-SP1 processing [66]. The significance of this observation is described in more detail below.

### 2.3. Structural Implications of MI Binding and Resistance

The effects of MI binding on Gag structure and the behavior of MI-resistant mutants in particle assembly and maturation have provided insights into the roles of CA and SP1 in the late stages of virus replication. Early thin-section transmission EM data indicated that particles produced in the presence of MIs were morphologically aberrant; they contained an electron-dense aggregate of material, often acentrically located in the virion, and a crescent of electron density along the edge of the particle [56] (see below). At the resolution provided by thin-section transmission EM, this virion morphology resembled that described earlier for particles produced by the so-called CA5 mutant, in which the CA-SP1 cleavage site had been inactivated by mutation [74]. However, higher-resolution data obtained by cryo-ET and subvolume averaging revealed important differences between MI-treated and CA5 virions: in particles produced in the presence of MI, the crescent of electron density displayed the structural properties of the immature Gag lattice. In contrast, CA5 particles contained a thin, mature-like Gag layer [75]. These results suggested that MIs stabilize the immature Gag lattice. Indeed, when CA5 particles were produced in the presence of MIs (either BVM or PF96), the immature Gag lattice was stabilized and did not convert to the mature-like lattice [24]. Interestingly, the SP1-T8I mutation, which rescues MI-dependent Gag mutants and blocks CA-SP1 processing [59,66], also displayed the stabilized, immature Gag lattice indistinguishable from that induced by MI binding [76]. These findings led to the hypothesis that MI treatment and the SP1-T8I mutation block CA-SP1 processing and maturation by stabilizing the immature Gag lattice. Recent structural studies have shed significant light on this hypothesis and sparked new topics of study linked to this key region of Gag (Figure 5).

Structural studies have provided important insights into the mechanism of action of HIV-1 MIs (Figure 5). Investigations into the mechanism of action of HIV-1 MIs and structural studies aimed at understanding the importance of the CA-SP1 region of Gag in assembly and maturation are fundamentally linked. Data obtained in a variety of experimental systems suggested that, at least under certain conditions, the CA-SP1 boundary region adopts a helical conformation [77,78,79]. An early cryo-ET study proposed that the CA-SP1 boundary region forms a six-helix bundle in the immature particle [80]. However, the highly dynamic nature of this putative helical bundle initially precluded high-resolution imaging. More recently, structural analyses, performed by X-ray crystallography, cryo-ET, and NMR, have confirmed the presence of a six-helix bundle spanning the CA-SP1 junction [21,22] and have provided evidence that MIs bind in a central channel inside the bundle [21,22,81]. The SP1-T8I mutation, which phenocopies the effect of MIs in blocking CA-SP1 processing, stabilizes the CA-SP1 helix putatively extending the six-helix bundle [20]. Consistent with this hypothesis, work presented at the 2020 Cold Spring Harbor Retroviruses meeting demonstrated that the SP1-T8I mutation induces an extension of the six-helix bundle as far as the NC domain (Peijun Zhang, personal communication) [82]. Furthermore, mutations in the CA MHR disrupt immature lattice assembly [21], presumably by destabilizing the six-helix bundle. Strikingly, in the fully folded six-helix bundle, the CA-SP1 cleavage site is buried and is thus inaccessible to PR [21,22]. These observations imply that the six-helix bundle must unfold to some extent to allow access by PR. Collectively, these findings give rise to the following working model for CA-SP1 processing and MI activity (Figure 5): the CA-SP1 boundary region is in a dynamic equilibrium between a folded six-helix bundle, in which the CA-SP1 cleavage site is inaccessible, and a more disordered conformation in which PR can gain access to the cleavage site. MIs stabilize the six-helix bundle, thereby preventing CA-SP1 cleavage by limiting the conformational changes in the bundle needed for PR access. Some of the MI-resistance mutations (e.g., those in the MHR and at SP1 residue 3) offset the stabilizing effect of MI binding by destabilizing the CA-SP1 boundary region. These mutants thus require the presence of the MI to assemble particles, making them compound dependent. Finally, the SP1-T8I mutation acts like a MI by stabilizing the bundle, blocking CA-SP1 processing, and rescuing these compound-dependent mutants (Figure 5). The studies that have contributed to this working model also implicate the destabilization of the six-helix bundle as the “switch” that drives the conversion of structurally immature CA to structurally mature CA during maturation. Therefore, MIs bind at the epicenter of a structural and proteolytic transition zone required for virus maturation and infectivity, making them valuable probes to study these key events.

Although work over the past several years has greatly increased our understanding of MI activity, several important questions remain. A recent NMR study demonstrated that BVM-induced changes in the structure and dynamics of the six-helix bundle of spherical virus-like particles (VLPs) were surprisingly subtle, raising questions about the mechanism by which MIs stabilize this region [83]. The dynamic nature of the CA-SP1 boundary continues to present challenges for defining the atomic-level structure of this region. The highest-resolution structure to date [81], obtained by micro-electron diffraction, provides a 2.9 Å view of BVM docked in the CA-SP1 six-helix bundle. BVM appears to be oriented with the dimethyl succinyl moiety facing the CA side of the bundle, which it stabilizes via both electrostatic and hydrophobic interactions. At this resolution, individual contacts between the compound and amino acid side chains are difficult to resolve. Precisely, how MIs interact with their target is a crucial question in understanding the basis for the greatly improved potency and breadth of activity of second-generation BVM analogs. Although, as mentioned above, it is likely that BVM and PF96 bind in a similar fashion, the low solubility of PF96 has stymied attempts to directly demonstrate this. Similarly, recent characterization of other second-generation MIs has provided further evidence of six-helix bundle binding and stabilization [84,85,86]. Resistance to MIs appears to arise via several mechanisms: loss of compound binding, compound dependency, and increased rates of CA-SP1 processing [59,63,66,87,88]. Further structural information will be necessary to elucidate the molecular mechanisms driving MI activity and six-helix bundle stability. One recent discovery that may shed significant light on these mechanisms is the identification of IP6 as a host cofactor for HIV-1 replication [49,50,51]. As mentioned above, IP6 interacts with two lysine rings (formed by CA-K158 and K227) just above the six-helix bundle that serves as the binding site for MIs [49] (Figure 4). In vitro, IP6 dramatically increases the kinetics of Gag assembly [89] and promotes the assembly of immature VLPs under conditions that favor the assembly of the mature lattice [49]. Furthermore, IP6 inhibits CA-SP1 proteolysis in a manner that is additive or synergistic with BVM in vitro [89]. These findings suggest that IP6 promotes assembly of the immature Gag lattice by stabilizing the six-helix bundle in a BVM-independent manner, indicating that IP6 does not compete with BVM for binding. Studies aimed at elucidating the relationship between IP6, MIs, and six-helix bundle stabilization in the context of assembly and maturation are ongoing.

### 2.4. Clinical Development of BVM and Second-Generation BVM Analogs

The high level of antiviral activity of BVM in vitro, coupled with positive preclinical safety and pharmacokinetics results [90], justified moving BVM into clinical testing in the mid-2000s. The phase IIa clinical trials produced mixed results; robust antiviral activity, as measured by a 1–2 log decline in viral loads over a 2-week treatment period, was observed in ~50% of participants, whereas significant reductions in viral loads were not observed in the remaining treated individuals [91,92,93]. Obtained data, both in vivo and in vitro, established that lack of response was associated with naturally occurring polymorphisms near the C-terminus of CA and within SP1 [87,91]. In particular, the SP1-V7A (Gag-V370A) polymorphism, present in a high percentage of HIV-1 isolates, reduced the susceptibility of HIV-1 to BVM [87,91]. The inability of BVM to effectively suppress the replication of V7A-containing isolates of HIV-1, together with its high plasma protein-binding properties, led to the discontinuation of BVM’s clinical development.

Over the past decade, several groups have conducted medicinal chemistry campaigns to identify analogs of BVM that overcome the loss of activity associated with polymorphisms in the CA-SP1 boundary region [94,95,96,97,98]. Modification of BVM at the carbon-3 (C-3) and C-28 positions (Table 1) has resulted in several “second-generation” MIs that retain antiviral potency against strains of HIV-1 that are not effectively suppressed by BVM [85,99,100]. Urano and colleagues [88] selected for resistance to some of these second-generation inhibitors in the context of both subtype B and C viral isolates. As with BVM and PF96, resistance mutations were observed near the CA-SP1 junction and in the MHR, although some novel SP1 mutations were observed in the subtype C selections. Similar results were obtained for the BVM derivative EP-39, though in silico docking experiments suggested that this compound may interact differently with the six-helix bundle [86]. Although high protein binding and poor solubility continue to present challenges for this class of molecules, the second-generation molecules display improvements in these properties relative to BVM. The BVM analog GSK-3532795/BMS-955176, was recently taken into clinical trials with largely favorable outcomes in terms of its ability to suppress viral replication [84,101,102,103]. However, clinical development of this compound was halted because of gastrointestinal intolerability and the emergence of the SP1-A1V resistance mutation, which is often observed with this class of molecules in vitro [103]. Additional molecules are currently being tested.

## 3. Targeting CA to Disrupt Capsid Assembly and Stability

In addition to blocking CA-SP1 processing, HIV-1 maturation can also be targeted by disrupting the assembly of the capsid from individual CA monomers. The discovery of inhibitors displaying this activity has been achieved via the implementation of a diverse set of experimental assays and screens. In vitro and in silico screens for CA binding, mechanism-blind screens for inhibitors of HIV-1 replication, and functional assays that assess the inhibition of CA aggregation or the assembly of mature-like CA-NC tubes have contributed to the identification of CA-based inhibitors. Below, we will review the activity of these compounds and insights into HIV-1 biology that their study has provided.

### 3.1. Identification of Several Classes of HIV-1 CA-Based Inhibitors in Antiviral Screens

*CAP-1:* In 2003, the first CA inhibitor, N-(3-chloro-4-methylphenyl)-N0-(2-[((5-[(dimethylamino)-methyl]-2-furyl)-methyl)-sulfanyl]-ethyl) urea or CAP-1 (Table 1) was identified in a computational screen of public chemical libraries for small molecules that were predicted to interact with CA. CAP-1 was shown to bind to the CA-NTD with weak affinity (~800 µM), slowed the in vitro assembly of CA into mature-like tubes, and reduced the infectivity of released particles by 95%. Virus production was not affected by CAP-1 treatment; however, EM analysis revealed that particles released from CAP-1-treated cells showed increased size heterogeneity and did not contain capsids [68]. This phenotype mirrored that of previously described CA mutants designed to abrogate CA–CA interactions required for mature capsid assembly [26,104,105], suggesting that CAP-1 blocks viral infectivity by inhibiting the assembly of mature capsids [68]. A follow-up study determined the CAP-1 binding site using a combination of X-ray crystallography and NMR. The authors generated a model of CAP-1 bound to the CA-NTD at the apex of a 5-helix bundle composed of CA helices 1, 2, 3, 4, and 7. CAP-1 was shown to induce significant conformational changes at its binding site, resulting in the displacement of three aromatic side chains (F32, H62, and Y145). It has been hypothesized that the conformational changes in this region could disturb intermolecular CA-NTD/CA-CTD (NTD-CTD) interactions crucial for the formation of mature CA hexamers [33,106]. However, mutations conferring resistance to this compound, which could shed light on CAP-1 binding and its antiviral mechanism, have not been reported.

*CAI/NYAD-1:* A 2005 phage display screen identified a 12-amino-acid peptide, CAI (sequence ITFEDLLDYYGP; Table 1), that effectively inhibits the assembly of both immature VLPs and mature CA tubes and spheres in vitro [69]. A crystal structure of CAI in complex with the CA-CTD was reported in an accompanying manuscript [107]. This analysis showed that the α-helical CAI converts the CA-CTD from a 4-helix into a 5-helix structure mainly through interactions with helices 8, 9, and 11 of the CA-CTD. Although the CAI binding site is near the CA-CTD dimer interface, NMR rotational correlation times suggested that the CA-CTD was still able to dimerize when bound to CAI [69]. However, the buried surface area at the dimer interface was much lower in the CA-CTD/CAI complex compared to the unliganded dimer [107]. This suggests that while CAI does not disrupt CA-CTD dimerization, it may induce the formation of a distorted dimer that could be nonfunctional during assembly [69,107] as proper CA-CTD dimerization is crucial for the assembly of both immature and mature particles [108,109,110,111]. Further studies employed different modes of hydrocarbon stapling to generate improved CAI-derived peptide inhibitors with enhanced cell permeability [112,113]. One of these peptides, NYAD-1, showed broad-spectrum activity against HIV-1 in culture, with IC_50_ values ranging from 4 to 21 µM. Furthermore, NYAD-1 retained the ability to block immature and mature CA assembly in both cell-free and cell-based systems and impaired virus release and particle infectivity [113]. Additionally, NYAD-1 and other similar NYAD-series peptides exhibited antiviral activity during early stages of the virus replication cycle. Mutations that conferred resistance to these peptides were selected and mapped to gp120, suggesting that the peptides may inhibit viral replication via multiple, unrelated mechanisms [112,113].

*PF74:* PF-3450074 (PF74) was first reported by Pfizer in 2010 as part of a screen for inhibitors of HIV-1 replication (Table 1) [70]. A high-resolution (1.8 Å) crystal structure revealed that PF74 bound with low-µM affinity to a pocket within the CA-NTD formed between helices 3, 4, 5, and 7. Later, PF74 was shown to bind to an intermolecular interface between the CA-NTD of one subunit and the CA-CTD of an adjacent subunit within the mature CA hexamer [32,47,48]. PF74 was reported to stabilize in vitro-assembled CA-NC complexes [114] and enhance the rate of CA multimerization [70]. Somewhat paradoxically, however, PF74 also facilitated the uncoating of HIV-1 capsids in cell-free and cell-based assays [115]. Furthermore, high concentrations (7–10 µM) of PF74 inhibited infection via dual mechanisms: an early reverse transcription block and, late in the replication cycle, disruption of mature capsid formation [70,115]. PF74-mediated destabilization of the HIV-1 capsid is supported by the observation that CA mutants exhibiting hyperstabilized cores were resistant to PF74, whereas CA mutants exhibiting destabilized cores were more sensitive to PF74 treatment [39,115,116]. However, recent studies suggest that PF74 may perturb capsid uncoating by hyperstabilizing the capsid [117,118]. Future studies addressing these seemingly conflicting observations will continue to shed light on the precise mechanism of PF74 inhibition.

Remarkably, PF74-mediated inhibition of early post-entry steps was shown to be bimodal, dependent on compound concentration. At 2 µM, PF74 inhibits HIV-1 infectivity in target cells without disrupting reverse transcription and without decreasing the number of RTCs and PICs in the cytoplasm of infected cells [119]. At concentrations above 10 µM, PF74 inhibits reverse transcription and destabilizes viral cores in infected cells [43,120,121]. PF74′s activity at lower concentrations was shown to be dependent on its ability to reduce nuclear entry by disrupting interactions between CA and host factors required for nuclear entry [43,121]. Although PF74 has been a valuable probe for studying post-entry events, its antiviral potency is relatively low and it exhibits extremely poor metabolic stability [122]. Recent studies addressing these issues have resulted in the development of modified compounds with improved potency and metabolic stability [123,124]. Compounds developed by Gilead Sciences based on the PF74 scaffold have shown tremendous therapeutic potential and are currently performing well in initial human clinical trials [71,125]. A more detailed discussion of these compounds will be provided below.

*Benzoimidazoles/Compound 1:* In 2012, a screen for compounds that blocked the in vitro assembly of CA-NC tubes identified two new classes of CA inhibitor: benzodiazepines (BDs) and benzimidazoles (BMs). These compounds bind deeply into the CAP-1 binding pocket, resulting in significantly increased potency (EC_50_ < 1 µM) relative to CAP-1. Interestingly, BD compounds block virus assembly and release, whereas BM compounds do not affect particle production but block the formation of mature capsids in released particles [126]. Optimization of the BM series resulted in the discovery of a new compound (compound 1 or C1) that binds to a novel pocket within the CA-NTD and enhances the crystallization of the CA-NTD with BM and BD compounds bound to the CAP-1 binding site [127,128]. C1 binds in a shallow pocket near the base of the CypA loop (connecting helices 4 and 5) and the loop that connects helices 6 and 7 [128]. A later study showed that C1 blocks viral infectivity by disrupting the assembly of mature capsids during maturation. This effect was defined by a significant increase in the number of eccentric particles produced from cells treated with C1 in comparison to particles produced from untreated cells [129]. This phenotype recapitulates that of the allosteric integrase inhibitor (ALLINI) BI-D (see below), suggesting that C1 may target additional viral proteins. Furthermore, engineered disulfide cross-linking experiments suggested that C1 may disrupt the threefold interhexameric interface during CA assembly. Additional studies will be necessary to thoroughly define the mechanism by which C1 disrupts particle maturation.

In addition to HIV-1 CA inhibitors that target viral maturation, a number of other notable CA inhibitors have been developed in the last decade that predominantly or exclusively interfere with early events in the HIV-1 replication cycle. In 2010, coumermycin A1 (C-A1), a DNA gyrase inhibitor, was shown to have antiretroviral activity via a dual mechanism; it impaired viral gene expression by inhibiting the dimerization of heat-shock protein 90 (Hsp-90), but also showed an independent ability to block HIV-1 integration. A single mutation within CA conferred resistance to the integration block, suggesting that CA was indeed the target of this compound during HIV-1 infection [130]. In 2012, a hybrid structure-based virtual screen for small molecules that could bind to the intermolecular NTD–CTD interface identified CK206, which inhibits HIV-1 in single and multicycle replication assays. Optimization of this small molecule led to the discovery of I-XW-053 and ultimately compound 34, which blocks HIV-1 replication in primary PBMCs by inhibiting the completion of reverse transcription [131,132]. No binding site or resistance information has been reported for these inhibitors making their precise target uncertain [132]. In 2013, pyrrolopyrazolone compounds BI-1 and BI-2 were identified in a cell-based screen for inhibitors of single-cycle HIV-1 infectivity [133]. BI-2 binds to the PF74 site on the CA-NTD, increases the rate of CA-NC tube assembly in vitro, and destabilizes capsids in HIV-1-infected cells [133,134]. However, unlike PF74, BI-2 does not inhibit the late events in the HIV-1 replication cycle [115,134]. Finally, ebselen, an organoselenium compound, was identified in a screen for CA-dimerization inhibitors. This compound is unique among CA inhibitors, in that it covalently binds to the CA-CTD and inhibits early events by hyperstabilizing capsids during infection [135].

Very recently, screens for HIV-1 CA inhibitors have led to the discovery of 6a-9, one of a series of 4-phenyl-1H-1,2,3-triazole phenylalanine derivatives with antiviral activity similar to that of PF74 [124,136]. Additionally, a screen for natural products that bind to the CA-CTD yielded two linear diarylheptanoids, rubranol and hirsutanonol, that bind to the CA-CTD and allosterically inhibit CA assembly in vitro [137]. Further studies aimed at discerning the mechanism of action of each of these classes of compound will be required to assess their therapeutic potential.

### 3.2. Structural Implications of CA Inhibitor Binding

The versatile CA protein plays key structural roles in the assembly of the immature Gag lattice in the cell and the assembly of the viral capsid during maturation. As progress has been made in developing CA inhibitors, our understanding of CA structure in the context of its fully assembled complexes has increased. The cross-talk between the fields of CA inhibitor development and CA structural biology has provided tremendous insight into the intrahexameric and interhexameric CA–CA interfaces that are responsible for proper assembly and stability of viral cores.

**Figure 6:**
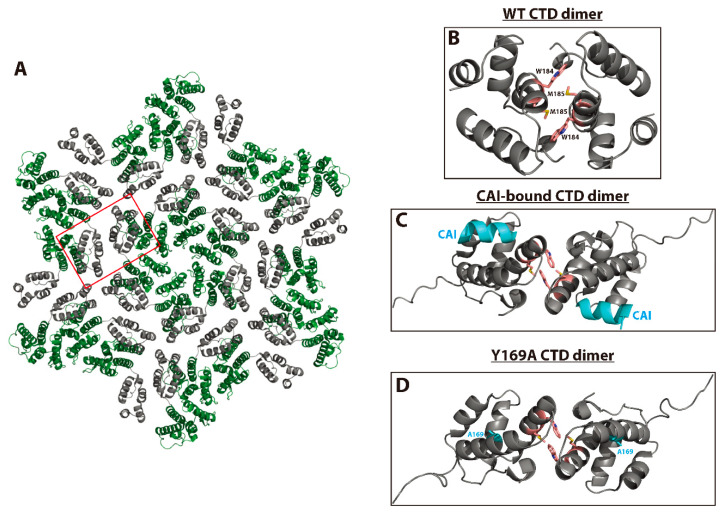
CAI induces aberrant dimer formation. (**A**) CA-CTD/CA-CTD dimerization within the mature CA lattice (red box) is an important interhexameric contact that holds mature CA hexamers together (PDB accession 5MCX). (**B**) WT CA-CTD dimer formation is mediated by two key amino acid residues: W184 and M185 (PDB accession 1A43). (**C**) CAI-binding to the CA-CTD allosterically induces the formation of an aberrant dimer in which W184 and M185 shift positions relative to the WT dimer (PDB accession 2BUO). (**D**) The Y169A mutation (blue) phenocopies the effect of CAI on the conformation of the dimer interface (PDB accession 3DS2).

The intermolecular CA-CTD/CA-CTD (CTD–CTD) interface is crucial for the assembly of both the immature Gag lattice and mature CA lattice due to its ability to dimerize and link hexamer subunits [108,109,110,111,138,139] (Figure 6A). As mentioned above, CAI binding to the CA-CTD causes an allosteric change in the dimer interface resulting in a dimer with a significantly decreased buried surface area compared to the unbound form [107] (Figure 6B,C). A follow-up study identified mutations within the CAI docking site (Y169A, L211A, and L211S) that decrease CAI binding and block in vitro assembly of CA and CA-NC tubes into mature-like particles. These mutants demonstrated aberrant core morphologies in tissue culture and were deficient in viral infectivity. Remarkably, crystal structures of these assembly-deficient CA-CTD mutants displayed allosteric changes that were very similar to those induced by CAI when bound to the wild-type CA-CTD [140] (Figure 6C,D). This includes a repositioning of the W184 and M185 side chains, which play a key role in WT CA dimer formation. These results underscore the crucial nature of the CA-CTD dimer interface as a structural element driving the proper assembly of mature capsids and suggest that even structural changes in this region that do not prevent dimer formation can have significant consequences for assembly and maturation.

**Figure 7:**
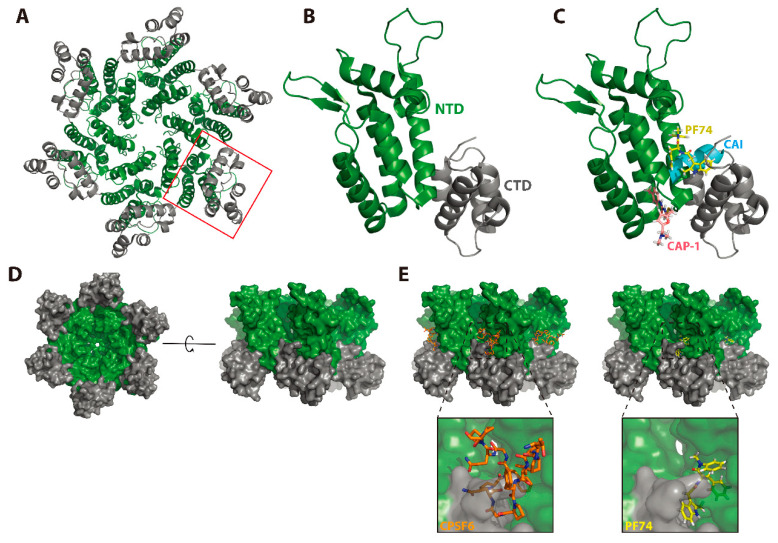
PF74 and other inhibitors perturb the CA-NTD/CA-CTD CA interface. (**A**,**B**) The intermolecular CA-NTD/CA-CTD interface (red box) contains key interactions stabilizing individual CA hexamers (PDB accessions 5MCX). (**C**) Multiple CA inhibitors capable of blocking capsid condensation during maturation bind to and potentially perturb this interface (PDB accessions 5MCX, 4QNB, 2BUO, and 2JPR). (**D**) Space-filling model of the mature CA hexamer (PDB accession 4WYM). (**E**) CA hexamer binding models for a peptide derived from CPSF6 and the CA inhibitor PF74 (PDB accession 4WYM and 4QBN).

The intermolecular CA-NTD/CA-CTD (NTD–CTD) interface between adjacent CA molecules within mature CA hexamers also plays a key role in capsid assembly and maturation (Figure 7A,B). In the early-2000s, numerous studies suggested that intermolecular interactions between the CA-NTD and CA-CTD occur in vitro and in vivo [109,141] and may be important for proper assembly of mature CA hexamers [142]. A CAP-1-bound model of the CA-NTD exhibited interesting conformational changes in the putative CAP-1 binding pocket and suggested that this inhibitor may disrupt the NTD–CTD interface (Figure 7C). Specifically, the Phe-32 side chain of the CA-NTD was displaced by approximately 6 Å from a buried state to a solvent-exposed state, causing a further displacement of the Tyr-145 side chain and a loss of electron density of the His-62 side chain. These conformational changes were predicted to facilitate CAP-1 binding via an induced fit mechanism in the pocket previously occupied by Phe-32 [106]. Furthermore, a low-resolution structure of the mature CA hexamer published just before strongly suggested that CAP-1 binding may disrupt the NTD–CTD intermolecular interface [139]. In 2009, the first high-resolution structure of the mature CA hexamer confirmed this prediction, demonstrating that the conformational changes required for CAP-1 binding could also disrupt a network of polar residues (Gln-63, Arg-162, and Asp-166) required to stabilize a key loop involved in the formation of the NTD–CTD interface [33]. This study also addressed the binding of CAI to the CA-CTD. Superposition of the CAI/CA-CTD complex with the NTD–CTD interface showed that binding of CAI to the CA-CTD occludes its interaction with the NTD (Figure 7C), suggesting that CAI could perturb mature CA assembly via multiple mechanisms. The NTD–CTD interface within CA hexamers is, therefore, clearly a promising target for the development of anti-HIV-1 compounds. In fact, other CA inhibitors such as BI-2, PF74, and the Gilead compounds discussed below also bind to the NTD–CTD interface (Figure 7C); however, the main antiviral activity of these inhibitors is exerted during early, post-entry events. These studies again illustrate the utility of small-molecule CA inhibitors as probes to understand the structure and function of their target.

### 3.3. PF74 Offers Insights into Post-Entry Events

The molecular basis for early post-entry events in the HIV-1 replication cycle is a focus of ongoing investigation. Recently, CA, and the capsids this protein forms, have been increasingly implicated in the regulation of key post-entry steps such as reverse transcription [39,115,131,132], cytoplasmic trafficking [143,144,145,146,147], nuclear entry [35,38,42,43,148,149,150,151], innate immune recognition [152,153], and integration of viral DNA [44,121,154,155]. These findings have been accompanied by the development of HIV-1 inhibitors that target incoming capsids and block crucial post-entry steps. Specifically, the bimodal mechanism of action by which PF74 inhibits these early steps (discussed above) has made this compound a useful chemical probe for studying post-entry events during HIV-1 infection. Interestingly, the PF74 binding site on viral capsids is shared by two host factors that have been implicated in nuclear entry of RTCs and integration of the viral DNA: cleavage and polyadenylation specificity factor 6 (CPSF6) and nucleoporin 153 (Nup153) [47,48,153,156] (Figure 7D,E). Because the PF74 binding site is lost upon disassembly of the CA hexamers, interactions involving this compound can only take place while the capsid is still in an intact or at least in semi-intact state [48]. Similarly, the interaction between the capsid and Nup153, a nuclear pore component, indicates that the capsid docks with the nuclear envelope in a largely assembled state. Additionally, the interaction between viral capsids and CPSF6 has also been implicated in nuclear entry [148,157] and nuclear trafficking of PICs to sites of integration [44]. A 2019 study demonstrated a role for CA during a post-nuclear-entry step, as PF74 was shown to inhibit PIC-associated integration in a capsid-dependent manner. These results suggest a role for at least partially assembled CA in the nucleus. Indeed, a 2020 study using a live-cell imaging approach and direct CA labeling found that intact or nearly intact viral capsids enter the nucleus via a CPSF6-dependent mechanism. Viral capsids in the nucleus were sensitive to PF74 treatment, suggesting that intact CA hexamers were present in these complexes. Interestingly, CA mutants (N74D and A77V) deficient in CPSF6 binding [40,158] exhibited prolonged docking, and ultimately uncoating, at the nuclear envelope. Furthermore, these mutants have been shown to integrate in gene-sparse heterochromatin near the nuclear envelope while viral capsids that uncoated in the nucleus integrated into gene-rich euchromatin [37]. These results parallel previous studies reporting that HIV-1 can utilize separate mechanisms of nuclear import dependent on the CPSF6 interaction [40] and that CPSF6 can direct PICs to specific sites within the host chromatin [44]. These results also provide context for a 2015 study [156] that found that HIV-1 could escape PF74 treatment while still retaining partial PF74 binding. The PF74-resistant virus not only was unable to interact with CPSF6 or Nup153 but also exhibited decreased dependence on these host factors, suggesting that PF74 resistance could also be mediated by use of different nuclear import mechanisms. Over the past decade, a great deal of progress has been made in our understanding of early post-entry steps in the HIV-1 replication cycle. More questions remain, and PF74 and other CA-binding small molecules will continue to be key tools in future investigations.

### 3.4. Preclinical and Clinical Development of GS-CA1 and GS-6207

In 2019, Gilead Sciences reported preclinical data for a novel CA inhibitor, GS-CA1, that blocks multiple steps during HIV-1 replication at sub-nM concentrations (EC_50_ ~ 130 pM in PBMCs) (Table 1) [71]. Interestingly, GS-CA1 exhibits three different profiles of inhibition at early post-entry steps. At high concentrations (25 nM), it interferes with reverse transcription, analogous to the activity of the RT inhibitor efavirenz (EFV). At intermediate concentrations (5 nM), GS-CA1 reduces the formation of integrated proviruses and 2-LTR circles without affecting overall levels of HIV-1 cDNA, suggesting a block in nuclear import of viral cDNA. At low concentrations (0.5 nM), this compound inhibits integration without affecting levels of viral cDNA or 2-LTR circles, suggesting that nuclear entry and integration are disrupted by distinct mechanisms. In addition, GS-CA1 treatment of virus-producer cells inhibits virus production and maturation resulting in an increased percentage of particles exhibiting aberrant core morphologies. This phenotype was shown to correlate with a dramatic decrease in infectivity in a single-cycle infectivity assay. As expected, given its distinct mechanism of action, GS-CA1 retains its antiviral potency against HIV-1 variants resistant to multiple classes of approved ARVs. In vitro resistance studies identified seven amino acid positions on CA (L56, N57, M66, Q67, K70, N74, and T107) at which mutations conferred varying levels of resistance to GS-CA1 (2.3-fold to >100 fold). Mutations at these positions were associated with a significant fitness cost for the virus. Mapping these amino acid positions onto a structure of the CA hexamer indicated that GS-CA1 binds to the same pocket where PF74, CPSF6, and Nup153 bind. This is unsurprising given the similar antiviral phenotypes displayed by GS-CA1 and PF74, albeit with significantly lower potency for the latter compound [115,121]. One mutation, M166I, confers significant resistance to both early and late GS-CA1-mediated blocks, suggesting that the mechanism of resistance is simply a displacement of GS-CA1 from its CA binding site. Finally, GS-CA1 was evaluated for its activity in vivo. Remarkably, a single subcutaneous dose of this compound in humanized mice was sufficient for the mice to maintain a plasma concentration well above the compound EC_95_ (630 pM) for the length of the study (56 days). The majority of GS-CA1-treated mice sustained viral suppression throughout the study, whereas one animal experienced virologic failure after 12 weeks. This failure was associated with the emergence of two CA mutations: Q67H and K70R. In contrast, 80% of the mice treated with a 10-fold higher dose of the NNRTI Rilpivirine experienced virologic failure and returned to baseline viremia by week 10 of the study [71]. The excellent potential of this compound as a potent, long-acting ARV has led to the development of GS-6207 [72], the first-in-class CA inhibitor to enter clinical trials (Table 1).

The first-in-class CA inhibitor GS-6207 exhibits a similar potency, mechanism of action, and resistance profile to GS-CA1. Likewise, it is in the same structural class and demonstrates a similar pharmacological and metabolic profile [71,72]. A crystal structure of GS-6207 in complex with cross-linked mature CA hexamers indicates that it docks in the PF74 binding pocket at the NTD–CTD interface. GS-6207 was shown to be highly potent in both target (EC_50_ = 25 pM) and producer cells (EC_50_ = 439 pM), indicating that it actively inhibits HIV-1 replication at both early and late stages of the replication cycle. Indeed, G2-6207 treatment induces an increase in the percentage of virions displaying aberrant capsid morphology, suggesting that capsid assembly is a target. However, its activity during early events is its most promising characteristic. GS-6207 has been hypothesized to inhibit nuclear entry and viral DNA integration [72], perhaps by competing with the binding of host factors CPSF6 and Nup153, which bind at the same interface on the capsid (Figure 7E). Thus far, clinical trials involving this compound have returned promising outcomes. Specifically, the results of two phase I trials suggest that both single-dose subcutaneous and oral administrations of GS-6207 are safe, well-tolerated, and supportive of less-frequent dosing [72,159,160]. Additionally, a phase 1b trial demonstrated a clear dose–response in antiviral activity marked by a decline in plasma HIV-1 RNA by 1.35–2.2 log_10_ copies/mL in over 9 days [72]. Further evaluation of GS-6207 as a long-acting ARV is underway as two phase II clinical trials with a 6-month dosing interval have begun recently. These trials will focus separately on treatment-naïve (NCT04143594) and heavily treatment-experienced (NCT04150068) persons living with HIV [125,161]. Furthermore, recent work presented at the 2020 Cold Spring Harbor Retroviruses meeting suggests that the primary molecular mechanism of GS-6207 is similar to that recently proposed for PF74 (Mamuka Kvaratskhelia, personal communication) [162]; i.e., GS-6207 perturbs capsid uncoating in infected cells via stabilization of the mature CA hexamers. Further characterization of the antiviral activity of this potent, first-in-class compound may drive the continued development of similar therapies.

## 4. Targeting Integrase to Disrupt RNA Condensation and Mature Capsid Formation

### 4.1. IN Mutations can Affect Virion Morphogenesis

The mature IN protein comprises three domains: the N-terminal domain, the central catalytic core domain, and the C-terminal domain (reviewed in references [163,164]). Four molecules of IN bind to the ends of the newly synthesized viral DNA to form the stable synaptic complex (SSC; also known as the intasome) that mediates the integration of the viral DNA into target cellular DNA. Integration involves two sequential chemical reactions: 3′-end processing, during which 3′-OH groups are prepared via removal of two nucleotides at the 3′ ends of the viral DNA, and DNA strand transfer, whereby these terminal 3′-OH groups are covalently joined to 5′ phosphates at the ends of cleaved cellular target DNA. FDA-approved IN inhibitors block strand transfer and are, therefore, referred to as INSTIs.

**Figure 8:**
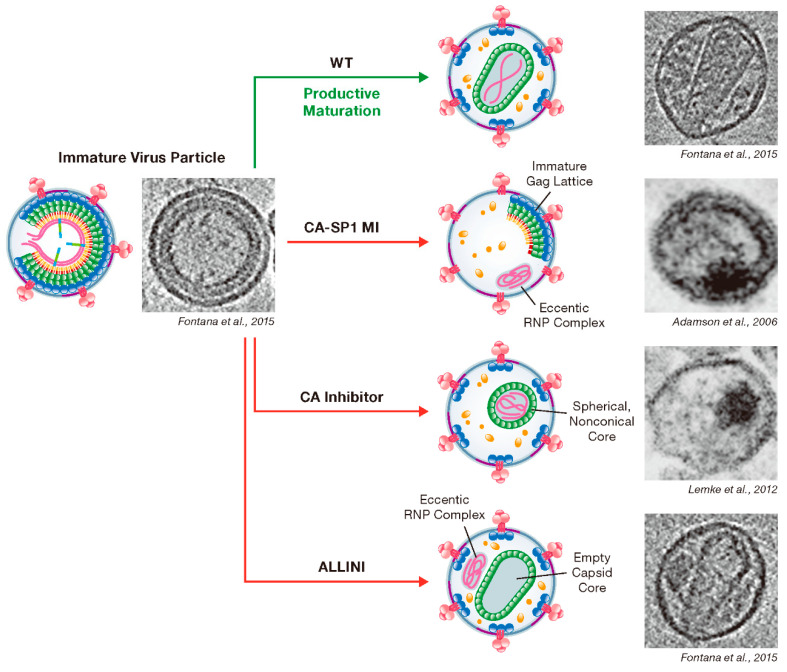
MIs exhibit diverse mechanisms of action. Productive maturation of HIV-1 particles can be inhibited by blocking multiple key steps such as CA-SP1 cleavage (CA-SP1 MIs), mature capsid assembly (CA inhibitors), and ribonucleoprotein (RNP) encapsidation (allosteric integrase inhibitors, ALLINIs). Representative micrographs, obtained by either thin section electron microscopy (EM) or cryo-electron tomography (cryo-ET), are shown, coupled with graphic representations of the EM data. Additional details are provided in the main text.

Early studies involving mutagenesis of HIV-1 IN identified residues in the enzyme required for viral DNA integration into the chromatin of the target cell (“class I” mutations) [165]. Mutational analyses also led to the surprising observation that some nonactive-site IN mutations blocked virion morphogenesis [28,166]. These so-called “class II” IN mutants produced eccentric particles that contained empty capsids and an electron-dense RNP condensate outside the capsid (Figure 8). The infectivity of these mutants was impaired at an early post-entry (reverse transcription) step [28,166]. Morphologically similar particles were also observed when a region spanning RT- and IN-coding regions was deleted from *pol*; these IN-deficient particles were also found to be defective in the formation of native RNA dimers [167]. Class II IN mutants could be rescued by providing a virion-packaged Vpr-IN fusion protein in trans, and IN catalytic activity was not required for this rescue [168]. These results collectively raised the possibility that IN plays a role in assembly and maturation distinct from its primary enzymatic function in viral DNA integration.

### 4.2. ALLINI-Treatment Mimics Class II IN Mutations

Studies in the mid-2000s identified lens epithelium-derived growth factor/p75 (LEDGF/p75) as a cellular cofactor that associates with the IN tetramer in the PIC and promotes integration by targeting the HIV-1 DNA to transcriptionally active genes [45,46,169,170,171,172,173]. LEDGF/p75 functions by binding to both target cell DNA and IN, thereby tethering the PIC to chromatin [174,175]. Compound-screening efforts led to the discovery of small molecules that target the LEDGF-binding site of IN, located at the IN dimer interface. These compounds have been referred to in the literature as LEDGF/p75-IN interaction site inhibitors (LEDGINs) [176], noncatalytic site IN inhibitors [177], integrase-LEDGF allosteric inhibitors (INLAIs), multimeric IN inhibitors (MINIs) [178], or allosteric IN inhibitors (ALLINIs) [31,178,179]. Initially, it was believed that the primary antiviral mechanism of action of these compounds was to block the binding between IN and LEDGF/p75 [176]; however, it was subsequently appreciated that they act mainly late in the HIV-1 replication cycle by inducing aberrant IN multimerization [180,181,182]. The key pharmacophores for the ALLINIs are a carboxylic acid and a tert-butoxy group projecting from a central aromatic ring system (Table 1). Structural analysis demonstrated that the ALLINI GSK1264 (Table 1) binds a site between the C-terminal domain and catalytic core of IN and bridges contacts between IN dimers, thereby inducing IN polymerization [73,183,184]. Strikingly, the morphology of particles produced in the presence of ALLINIs resembles that of the abovementioned class II IN mutants described many years previously [29,30,180,185]. The eccentric condensates located outside of the capsid in these particles were shown to have high NC content, confirming that ALLINIs and class II IN mutations impair viral RNP incorporation into capsids [29]. Furthermore, upon infection of new target cells, the viral RNA genome and IN from these ALLINI-treated eccentric particles are quickly degraded despite the presence of a separately active RT and assembled capsid [186].

The effect of class II IN mutations and ALLINI treatment on RNP condensation within the viral core prompted Kessl et al. to investigate whether IN plays a direct role in RNA condensation and particle maturation [31]. Indeed, cross-linking immunoprecipitation followed by sequencing (CLIP-seq) assays demonstrated that IN binds to the viral RNA within HIV-1 particles, exhibiting preferential interaction with specific structural elements in the viral RNA. This specific IN-RNA binding could be abrogated by treatment of virus-producing cells with ALLINIs. In vitro, IN binding to viral RNA induces the formation of RNA multimers that are bridged by IN. The RNA-binding activity of IN maps to several basic residues in the C-terminal domain of the protein. Mutation of these basic residues resulted in loss of IN-RNA binding and the production of virus particles with the class II phenotype in which the viral RNA did not condense properly within the capsid. Although the effect of the ALLINIs on proper core condensation has contributed to a greater understanding of the role of IN in particle maturation, several questions remain. Multimerization and vRNP binding are key properties required for IN function during maturation; however, the mechanism by which the viral RNP is localized *inside* the capsid is unclear. It is possible that IN could promote the formation of the capsid around the vRNP via a bridging interaction. Further investigation will be required to test this hypothesis [187].

### 4.3. Progress in the Development of ALLINIs as Therapeutics

At the 2020 Conference on Retroviruses and Opportunistic Infections (CROI), ST Pharm and collaborators at Emory University and the University of Colorado reported on STP0404, a safe and highly potent ALLINI [188]. In this study, STP0404 was shown to bind the LEDGF/p75 docking site on the HIV-1 IN dimer and exhibit low- to sub-nM EC_50_ values in culture. Furthermore, virus particles produced from STP0404-treated cells exhibited the same eccentric condensate that characterizes experimental ALLINI compounds. Two mutations within the inhibitor binding site (Y99H and A128T) conferred resistance to STP0404; however, raltegravir-resistant strains and natural HIV-1 variants that are resistant to other ALLINIs [189,190] were not resistant to STP0404. STP0404 also suppressed HIV-1 rebound in latently infected T-cells. Finally, STP0404 exhibited positive pharmacokinetic profiles and no toxicity issues in dogs and rats. STP0404 will move into phase 1 clinical development in 2020.

## 5. Concluding Remarks and Future Perspectives

Upon the assembly and release of immature HIV-1 virions from an infected cell, several key biochemical steps, collectively referred to as maturation, must be completed properly to generate fully infectious particles. First, the Gag and GagPol polyprotein precursors must be proteolytically processed by PR. Next, the released CA protein must undergo a structural conversion to facilitate its assembly into the mature lattice. Finally, CA must assemble into a stable core encapsidating the viral genome and the viral enzymes (RT and IN) required for the productive infection of a new target cell. In this review, we have broadly used the term “MI” to refer to small molecules that block any or all of these steps. Over the last decade, major advances have been made in elucidating the genetic, biochemical, and structural determinants of HIV-1 maturation. These advances have been accompanied by, and often informed, the identification and characterization of MIs. Despite the considerable progress outlined in this review, significant gaps remain in our understanding of the molecular mechanisms driving key steps during maturation. As we move forward in addressing these gaps, we can expect that MIs will continue to play an essential role in future studies.

A growing body of evidence indicates that IP6 plays a crucial role during HIV-1 assembly and maturation [49,50,51]. IP6′s ability to stabilize both Gag hexamers during immature lattice assembly and CA hexamers during mature capsid assembly highlights its importance throughout the maturation process. Furthermore, its binding site within Gag hexamers is very near the proposed binding site for MIs such as BVM and PF96. Therefore, IP6 and MIs that block CA-SP1 processing by stabilizing the six-helix bundle region of Gag hexamers may demonstrate some overlap in function or even compete with one another for binding. It seems likely that the continued study of these compounds will further elucidate the molecular mechanisms driving HIV-1 assembly and maturation and inform the development of more potent and better-targeted therapeutics. In particular, the development of molecules that inhibit CA-SP1 processing by binding competitively with IP6 could function similarly to current MIs with the added benefit of blocking IP6 packaging, effectively depriving the mature CA lattice of a key cofactor for its assembly. The structurally distinct nature of the BVM-derived molecules compared to PF96 suggest that the development of new classes of molecules that bind to the six-helix bundle is possible.

Another consideration for the development of novel MIs is the blocking of other Gag or Pol cleavage sites. Current MIs blocking CA-SP1 processing are effective because CA-SP1 cleavage is a key event in triggering the rearrangement of the liberated CA to facilitate mature capsid assembly. However, blocking other cleavage events may impede virus replication by different mechanisms. For example, previous studies have shown that the uncleaved MA-CA Gag fragment is a very strong dominant negative HIV inhibitor with the ability to induce aberrant and eccentric core morphology, block reverse transcription in target cells, and disrupt proper nuclear entry and integration [191,192]. However, the MA-CA junction is unstructured [193] and, therefore, may not be readily inhibited by small molecules. Another potential target is the SP1-NC cleavage site, which is the first site in Gag targeted by the viral PR during maturation. As discussed earlier, recent work presented at the 2020 Cold Spring Harbor Retroviruses meeting has described the effect of the SP1-T8I mutation (which is known to phenocopy MIs such as BVM and PF96 by stabilizing the six-helix bundle of Gag hexamers and inhibiting CA-SP1 processing) on the SP1-NC junction [82]. This work demonstrates that the SP1-T8I mutation causes the CA-SP1 junction helix to be extended through the N-terminal amino acid residues of the NC domain of Gag. Although there is currently no evidence that the SP1-T8I mutation blocks SP1-NC cleavage, it is tempting to apply the same logic used to generate the CA-SP1 model of MI inhibition to the SP1-NC junction. Perhaps molecules capable of stabilizing the SP1-NC junction (or even the entire CA-SP1-NC junction) would exhibit antiviral activity via a similar mechanism.

It could also be productive to consider other ways to induce the eccentric core phenotype seen in virions released from cells treated with ALLINIs. Perhaps compounds targeting the NC domain of Gag or the viral RNA genome could disrupt RNP condensation and/or encapsidation. Likewise, compounds targeting the packaging of the viral enzymes RT or IN could render properly formed capsids dead on arrival in the cytoplasm of target cells.

Ultimately, the antiviral strategy of maturation inhibition seems poised to remain a priority in the search for anti-HIV compounds. The diversity of viral mechanisms and components available to target during maturation generates a wide range of possibilities for novel antiviral compound development. In addition, such studies will further enhance our understanding of the genetic, biochemical, and structural mechanisms driving HIV-1 maturation.

## Figures and Tables

**Table 1:** Maturation inhibitors discussed in this review.

Compound	Structure	Target	Reference
Bevirimat (BVM)	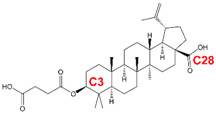	CA-SP1	Kashiwada et al. *J. Med. Chem.* 1996 [54].
PF-46396 (PF96)	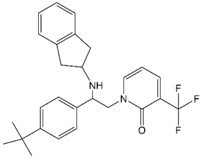	CA-SP1	Blair et al. *AAC* 2009 [65].
CAP-1	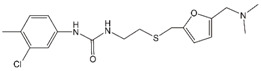	CA-NTD/CA-CTD interface	Tang et al. *J. Mol. Biol.* 2003 [68].
CAI	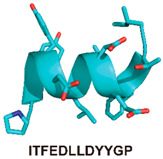	CA-NTD/CA-CTD interface; CA-CTD/CA-CTD interface	Sticht et al. *Nat. Struct. Biol.* 2005 [69].
PF74	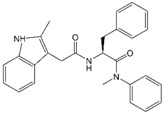	CA-NTD/CA-CTD interface	Blair et al. *PLoS Pathog.* 2010 [70].
GS-CA1	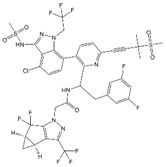	CA-NTD/CA-CTD interface	Yant et al. *Nat. Med.* 2019 [71].
GS-6207	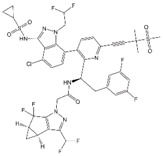	CA-NTD/CA-CTD interface	Link et al. *Nature* 2020 [72].
GSK1264	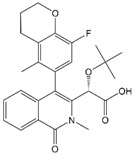	Integrase	Gupta et al. *J. Biol. Chem.* 2014 [73].
